# Cumulative rabbit anti-human thymocyte globulin dose to recipient weight during the peri-operative period is an independent risk factor for early postoperative urinary tract infection after kidney transplantation

**DOI:** 10.1080/0886022X.2024.2414841

**Published:** 2024-10-16

**Authors:** Shujuan Li, Ziyu Wang, Zhen Dong, Yanwei Cao, Hongyang Wang

**Affiliations:** Department of Kidney Transplantation, The Affiliated Hospital of Qingdao University, Qingdao, China

**Keywords:** Kidney transplantation, anti-human thymocyte globulin-Fresenius, urinary tract infection, dose, rejection

## Abstract

Anti-human thymocyte globulin-Fresenius (ATG-F) is frequently utilized to achieve successful induction for kidney transplantation recipients. This study aimed to examine the association between the ATG-F dose-to-recipient-weight ratio (ADR) and the risk of developing urinary tract infections (UTIs) following kidney transplantation. Data of kidney transplant recipients who underwent ATG-F-induction peri-operatively in a medical center were retrospectively collected, and the incidence of UTIs during the first postoperative year was also recorded. The risk of UTI associated with ADR was analyzed, and receiver operating characteristic curves were drawn to determine the optimal ADR, followed by Cox regression models. In total, 131 recipients were included, with an UTI incidence of 19.08% and a mean interval of 3.08 months. The optimal ADR was 6.34, involving 41 and 90 patients in the low ADR and high ADR groups, respectively. The UTI-free rate in the low ADR group was significantly higher than that in the high ADR group (*p* = 0.007). Cox regression analysis indicated that a high ADR independently increased the risk of UTI following kidney transplantation (hazard ratio: 5.306, 95% confidence interval: 1.243–22.660, *p* = 0.024). There was no significant difference in rejection rate between the high ADR and low ADR groups. In conclusion, a high ADR increased the incidence of early postoperative UTI among kidney transplant recipients.

## Introduction

Urinary tract infections (UTIs) are the most frequent complication following kidney transplantation [1]. The incidence of UTI in this population is influenced by factors, including age, sex, kidney function, and the dosage of immunomodulators for transplantation [2]. Anti-human thymocyte globulin-Fresenius (ATG-F), a polyclonal antibody generated by stimulating human T lymphocyte-like cell lines in rabbits, has exhibited tolerance-inducing effects. ATG-F is a frequently used antibody for achieving peri-operative immune induction during kidney transplantation, substantially lowering the risk of rejection and improving allograft survival [3]. However, the recommended standard dosage of ATG-F remains elusive. Prior studies demonstrated that the risk of acute rejection increased when ATG-F dosage was suboptimal; in contrast, the risk of postoperative infection was also elevated when the ATG-F dosage was extremely high [4–6]. When postoperative infections, such as UTI, occur in transplant recipients, the subsequent risk of progressive kidney function loss and mortality rises [7,8]. Kadon et al. demonstrated that high ATG-F doses significantly lowered the number of CD3+, CD4+, and CD8+ cells within five days after transplantation, predisposing these patients to early postoperative infections [9]. Our previous research indicated that a higher proportion of patients receiving ATG-F, as well as those administered higher doses of ATG-F, faced an increased risk of UTIs within the first postoperative year following kidney transplantation [10]. Other studies demonstrated that ATG-F could cause T lymphocyte depletion lasting for more than one year [11,12]. However, the association between ATG-F dose-to-weight ratio (ADR) and the risk of early postoperative UTIs has not been examined. Therefore, the present study aimed to evaluate the relationship between peri-operative ADR and the risk of developing UTIs following kidney transplantation.

## Methods

### Study subjects

Data of kidney transplant recipients who received a deceased donor (DD) kidney and were treated with ATG-F exclusively during induction in the perioperative period from July 2015 to July 2018 at the Department of Kidney Transplantation, Affiliated Hospital of Qingdao University (Qingdao, China) were retrospectively collected. The vast majority of organ donations in our transplant center come from DDs, with less than 5% from living donors. In addition, organs from living relative donors typically have a high compatibility with recipients, and Basiliximab is more commonly utilized for immune induction rather than ATG-F. All participants received only one kidney transplant and a triple immunosuppressive regimen postoperatively. FK506, MPA or MMF, and hormones (methylprednisolone) were administered on the first day after renal transplantation. The induction dose of ATG-F was 2 mg/kg on the day of surgery, and the highest prescribed dose was less than 200 mg. The subsequent ATG-F dose was 0.5–1.5 mg/kg during the first to the fourth days after surgery, rounded to the nearest vial size (100 mg), depending on the number of T cells. Supplementary Figure S1 presents total drug doses, the duration of ATG-F use, and the dose per day for ATG-F. The daily dosage of FK506 and MPA or MMF was approximately 0.1 mg/kg. The dose was adjusted according to the serum concentration of FK506 and maintained at 8–10 ng/mL during the first month, 6–8 ng/mL during the second to sixth months, and 5–7 ng/mL thereafter. The daily dosage of MMF was 1000 mg for patients weighing less than 70 kg and 1500 mg for those weighing 70 kg or more. The daily dosage of MPA was 720 mg for patients weighing less than 70 kg and 1080 mg for those weighing 70 kg or more. Methylprednisolone was administered at a dose of 16 mg daily starting on the third day post-operation, followed by gradual tapering to 4 mg on 90 days after surgery. The exclusion criteria were summarized as follows: (1) severe preoperative infections; (2) severe diseases, involving heart, brain, liver, lung, or other organs; (3) the presence of DD infection; (4) malignant tumors; and (5) other major complications (e.g., anastomotic fistula, ureterostenosis, ureteral obstruction) during the peri-operative period. This study was approved by the Medical Ethics Committee of Qingdao University (Approval No. QYFYWZLL 26483). All participants signed the informed consent form prior to enrollment.

### Data collection

Trained researchers recorded clinical data, including patient age, gender, diabetes mellitus status, the origin of end-stage kidney disease, the modality of dialysis prior to transplantation, postoperative immunomodulatory regimen, the duration of ureteral stent indwelling, postoperative serum tacrolimus concentration, and the timing of postoperative UTIs (if occurred). The diagnosis of UTIs was based on typical febrile symptoms (>38 °C), urgency, increased frequency of voiding, dysuria, suprapubic tenderness, burning sensation during voiding, and a positive urine culture (>100,000 microbial colony forming units per mL of urine) [13]. Once UTIs were diagnosed, the corresponding antibiotics were administered according to the antibiogram results.

Cumulative ATG-F dosage was also recorded during the peri-operative period, and ADR was calculated based on the ratio of cumulative ATG-F dosage relative to body weight during the peri-operative period. UTIs were diagnosed using the following criteria: midstream urine culture yielding a bacterial load ≥10^5^/mL, urine leukocytes >10 in midstream urine determined using a high-power field magnification, and the presence of typical symptoms of UTIs (frequency, urgency, dysuria, and fever) with pathogenic bacteria identified.

### Clinical follow-up and outcome determination

Participants were followed up at least monthly for more than one year. During follow-up, documentation included whether participants developed symptoms and signs of UTIs, whether urinalysis and urine cultures were performed, and whether urine cultures exhibited a bacteria load of ≥10^5^/mL. The incidence rates of pulmonary infection, BK virus infection, and cytomegalovirus infection were also recorded.

### ADR categorization

The receiver operating characteristic (ROC) curve was plotted based on the analytic results between ADR values and UTI, and the optimal cutoff ADR value was determined for ADR categorization. All participants were divided into low ADR and high ADR groups, according to the cutoff value.

### Statistical analysis

The statistical analysis was conducted through R 3.4.3 software. Data conforming to the normal distribution were expressed as mean ± standard deviation. Data that did not conform to the normal distribution were expressed as median with interquartile differences. Normally distributed data were compared using the *t*-test, and the non-parametric rank-sum test was employed otherwise for making comparison. Clinical data were compared between the low ADR and high ADR groups. The incidence of UTI within one year following kidney transplantation was compared between groups using Kaplan-Meier analysis. The relationship between cumulative ADR of allograft recipients and the risk of early UTI following kidney transplantation was also assessed. The log-rank test was used to compare the incidence of UTI between the low ADR and high ADR groups. The number of cases (percentage) was compared between the two groups using the Chi-square test. The risk factors for developing early UTI after kidney transplantation were assessed using univariate and multivariate Cox regression models. Variables with *p* < 0.1 were involved in the multivariate logistic regression analysis. Correlation analysis was carried out using the Spearman correlation coefficient. A two-tailed *p* < 0.05 was considered statistically significant.

## Results

A total of 131 kidney transplant recipients were analyzed, including 102 (77.86%) men and 29 (22.14%) women. None of these patients received combined kidney and pancreatic transplantation. Their mean age was 40.73 ± 10.46 years old. Among the 131 patients, 25 had a UTI. There were 7 cases of *Escherichia coli* infection with ampicillin, gentamicin, cotrimoxazole, cefazolin, and ceftazidime resistance; 6 cases of *Klebsiella pneumoniae* infection, with levofloxacin, ampicillin, aztreonam, ceftazidime, cefazolin, ciprofloxacin, cefazoline, sulbactam, cefepime, cotrimoxazole, piperacillin, tobramycin, and nitrofurantoin resistance; 4 cases of *Pseudomonas aeruginosa* infection, presented as nitrofurantoin and imipenem resistance; 2 cases of *Enterococcus faecalis* infection, with levofloxacin, ciprofloxacin, erythromycin, moxifloxacin, and tetracycline infection; 2 cases of *Stenotrophomonas maltophilia*, with no drug resistance; 1 case of *Enterococcus faecium*, with moxifloxacin, levofloxacin, ampicillin, ciprofloxacin, erythromycin, penicillin, nitrofurantoin, and tetracycline resistance; 1 case of golden grape infection, with clindamycin, erythromycin, penicillin, and oxacillin resistance; 1 case of *Staphylococcus aureus* infection, with ciprofloxacin, clindamycin, erythromycin, gentamicin, and oxacillin resistance; and 1 case of *Citrobacter freundii* infection, with cotrimoxazole resistance (Table S1).

**Figure 1.**
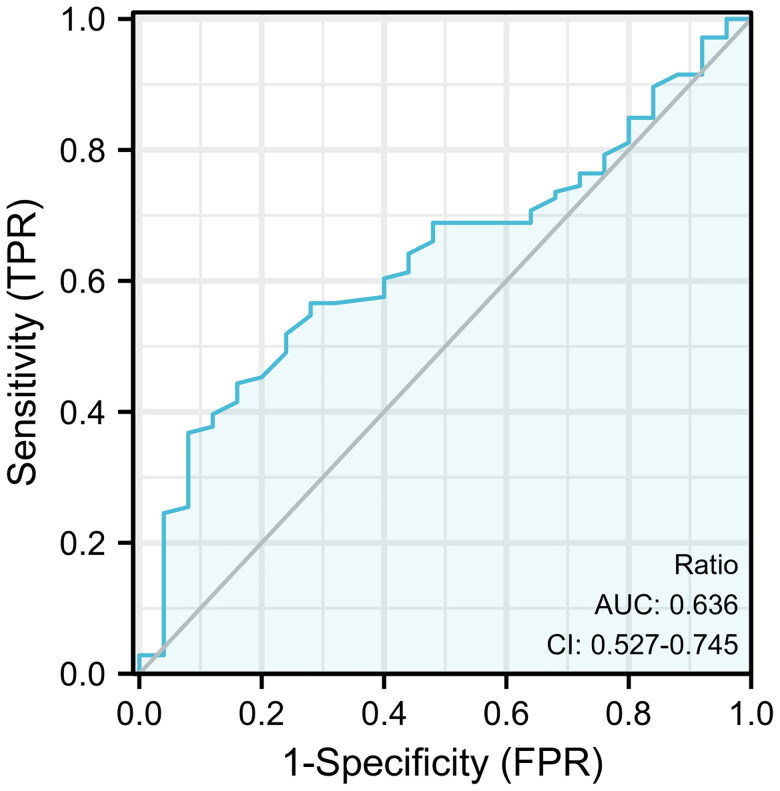
Receiver operating characteristic curves of early urinary tract infection probability after kidney transplantation, as predicted using the dose-to-weight ratio of rabbit anti-human thymocyte globulin-Fresenius.

The area under the ROC curve (AUC) for the analysis of the association between ADR (mg/kg) and incidence of UTI was 0.636 (95% confidence interval (CI): 0.527-0.745, *p* = 0.035). The maximum Youden index was 0.288, with an optimal cutoff value of 6.34 mg/kg. Moreover, 67 patients had premature discontinuation of ATG due to severe complications. The low ADR group (*n* = 41) was defined as those having an ADR lower than 6.34 mg/kg, and the high ADR group (*n* = 90) was defined as those having an ADR higher than 6.34 mg/kg (Figure 1).

**Figure 2.**
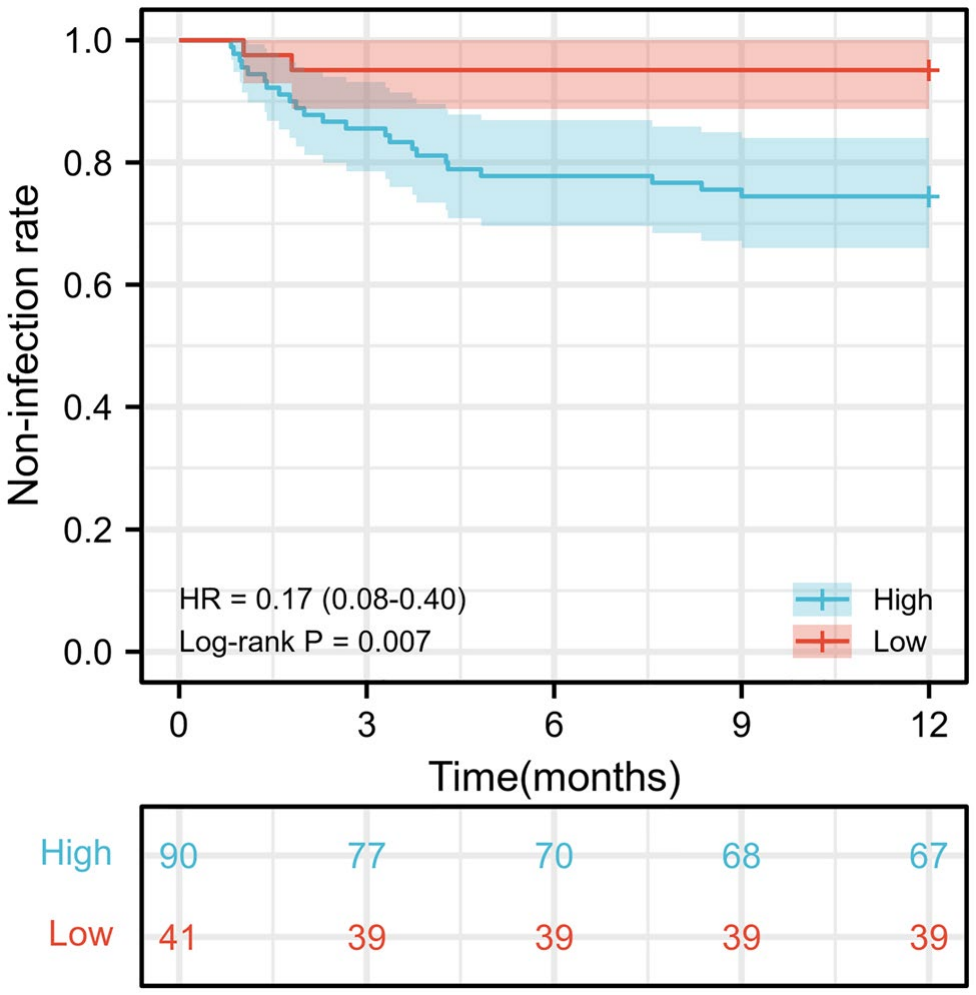
Kaplan-Meier event-free curves drawn according to rabbit anti-human thymocyte globulin-Fresenius dose-to-weight ratio of patients after kidney transplantation.

There was no significant difference between the low ADR and high ADR groups in gender (*p* = 0.064), age (*p* = 0.061), diabetes mellitus status (*p* = 0.655), origin of end-stage kidney disease (*p* = 0.125), pre-operative dialysis modality (*p* = 0.559), duration of ureteral stent indwelling (*p* = 0.972), type of immunomodulator used (*p* = 0.629), serum tacrolimus concentration at postoperative 1, 3, 6, and 12 months (all *p >* 0.05), pulmonary infection (*p* = 0.371), lymphocyte count (all *p > 0*.05), lymphocyte percentage (all *p > 0*.05), history of UTIs (*p* = 0.498) or history of urolithiasis (*p* = 0.181). However, the infection rates of BK virus (*p* = 0.037) and cytomegalovirus (*p* = 0.035) significantly differed between the high ADR and the low ADR groups (Table 1). No significant difference was noted between the two groups except for their ADR level (Table 2). The Kaplan-Meier event-free curves were plotted to compare the incidence rates of UTIs between the two groups (Figure 2). The UTI-free survival was significantly higher in the low ADR group than that in the high ADR group (the former vs. the latter, 95.12% vs. 74.44%, χ^2^=7.258, *p* = 0.007).

**Table 1.** Comparison of clinical features between the low ADR and high ADR groups.

Variables	Test cohort (*n* = 131)	Low dose-weight ratio (*n* = 41)	High dose-weight ratio (*n* = 90)	P
Gender:				0.064
Male (n, %)	102 (77.86)	36 (87.80)	66 (73.33)	
Female (n, %)	29 (22.14)	5 (12.20)	24 (26.67)	
Age (years):				0.061
≤40 (n, %)	67 (51.15)	16 (39.02)	51 (56.67)	
>40 (n, %)	64 (48.85)	25 (60.98)	39 (43.33)	
Diabetes:				0.655
Yes (n, %)	20 (15.26)	8 (19.51)	12 (13.33)	
No (n, %)	111 (84.73)	33 (80.49)	78 (86.67)	
Stents indwelling time (months):				0.972
≤1.86 (n, %)	61 (46.56)	19 (46.34)	42 (46.67)	
>1.86 (n, %)	70 (53.44)	22 (53.66)	48 (53.33)	
Type of dialysis:				0.559
hemodialysis (n, %)	109 (83.21)	35 (85.37)	74 (82.22)	
peritoneal dialysis (n, %)	22 (16.79)	6 (14.63)	16 (17.78)	
Immunosuppression regimen:				0.629
FK506 + MPA (n, %)	68 (51.91)	20 (48.78)	48 (53.34)	
FK506 + MMF (n, %)	63 (48.09)	21 (51.22)	42 (46.67)	
Indication for transplant:				0.125
CGN (n, %)	92 (70.23)	23 (56.10)	69 (76.67)	
IgAN (n, %)	11 (8.40)	5 (12.20)	6 (6.67)	
DN (n, %)	13 (9.92)	5 (12.20)	8 (8.89)	
HTN (n, %)	4 (3.05)	3 (7.32)	1 (1.11)	
PCKD (n, %)	4 (3.05)	2 (4.88)	2 (2.22)	
Others (n, %)	7 (5.34)	3 (7.32)	4 (4.44)	
FK506 concentration				
One month after surgery (μmol/L)	9.08 ± 1.90	9.13 ± 2.08	9.06 ± 1.82	0.845
Three months after surgery (μmol/L)	8.57 ± 1.59	8.44 ± 1.34	8.63 ± 1.71	0.530
Six months after surgery (μmol/L)	7.05 ± 1.73	7.37 ± 1.63	6.90 ± 1.76	0.153
Twelve months after surgery (μmol/L)	6.71 ± 1.65	6.42 ± 1.71	6.84 ± 1.62	0.232
Weight (kg)	65 (57.5, 75)	76.65 ± 14.45	62.16 ± 10.30	<0.001
Dose (mg)	500 (400, 500)	400 (400, 500)	500 (400, 500)	<0.001
Pulmonary infection	18 (13.74)	4 (9.76)	14 (15.56)	0.371
BK virus infection	31 (23.66)	5 (12.20)	26 (28.89)	0.037
Cytomegalovirus infection	35 (26.72)	6 (15.00)	29 (32.22)	0.035
Lymphocyte count (10^9^)				
Preoperative lymphocyte count (10^9^)	1.35 (1.05, 1.66)	1.29 (1.01, 1.75)	1.36 (1.08, 1.60)	0.689
Lymphocyte count at 1 day after surgery (10^9^)	0.13 (0.09, 0.23)	0.12 (0.10, 0.23)	0.14 (0.08, 0.23)	0.984
Lymphocyte count at 7 days after surgery (10^9^)	0.80 (0.57, 1.12)	0.84 (0.59, 1.16)	0.80 (0.56, 1.11)	0.464
Lymphocyte count at 30 days after surgery (10^9^)	0.90 (0.62, 1.39)	1.04 (0.58, 1.60)	0.87 (0.64, 1.30)	0.211
Lymphocyte count at 90 days after surgery (10^9^)	1.34 (0.93, 1.74)	1.41 (1.03, 1.98)	1.32 (0.85, 1.69)	0.126
Lymphocyte count at 180 days after surgery (10^9^)	1.54 (1.17, 1.97)	1.66 ± 0.55	1.51 (1.11, 1.87)	0.175
Lymphocyte count at 360 days after surgery (10^9^)	1.65 (1.21, 2.16)	1.77 (1.22, 2.04)	1.63 (1.21, 2.22)	0.911
lymphocyte percentage (%)				
Preoperative lymphocyte percentage (%)	21.2 (17. 7, 25.9)	21.45 ± 5.82	21.25 (17.06, 26.50)	0.768
Percentage of lymphocytes 1 day after surgery (%)	1.10 (0.80, 1.70)	1.10 (0.85, 1.75)	1.10 (0.70, 1.73)	0.583
Percentage of lymphocytes 7 days after surgery (%)	9.72 (7.80, 12.40)	10.70 (8.25, 13.30)	9.66 (6.90, 12.13)	0.067
Percentage of lymphocytes 30 days after surgery (%)	14 (10.30, 20.40)	15.00 (10.30, 22.70)	13.92 (10.02, 18.15)	0.336
Percentage of lymphocytes 90 days after surgery (%)	21.6 (17.00, 28.20)	24.16 ± 8.07	20.95 (15.68, 27.58)	0.091
Percentage of lymphocytes 180 days after surgery (%)	25.38 ± 8.83	26.47 ± 9.92	24.89 ± 8.30	0.340
Percentage of lymphocytes 360 days after surgery (%)	25.89 ± 8.55	25.66 ± 9.68	26.00 ± 8.03	0.834
History of urinary tract infections (n, %)	1 (0.01)	0 (0)	1 (0.01)	0.498
History of urolithiasis (n, %)	3 (0.02)	2 (0.05)	1 (0.01)	0.181

**Table 2.** Kaplan-Meier Analyses of the UTI-free rate of patients following kidney transplantation.

Prognostic parameters	Log-rank χ^2^	P
Age	0.723	0.125
Gender	3.465	0.063
Diabetes	0.05	0.824
Stents indwelling time	2.284	0.131
Immunosuppression regimen	<0.001	0.993
ADR	7.258	0.007

**Figure 3.**
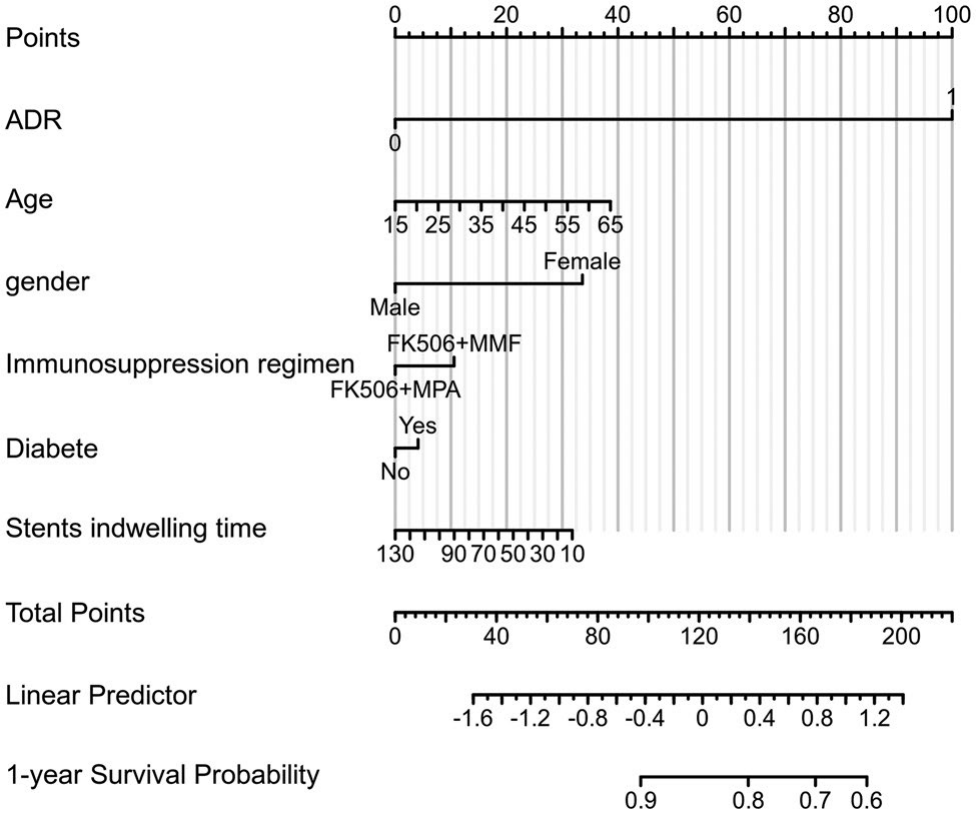
Nomogram for the prediction of 1-year early urinary tract infection incidence of patients following kidney transplantation.

**Figure 4.**
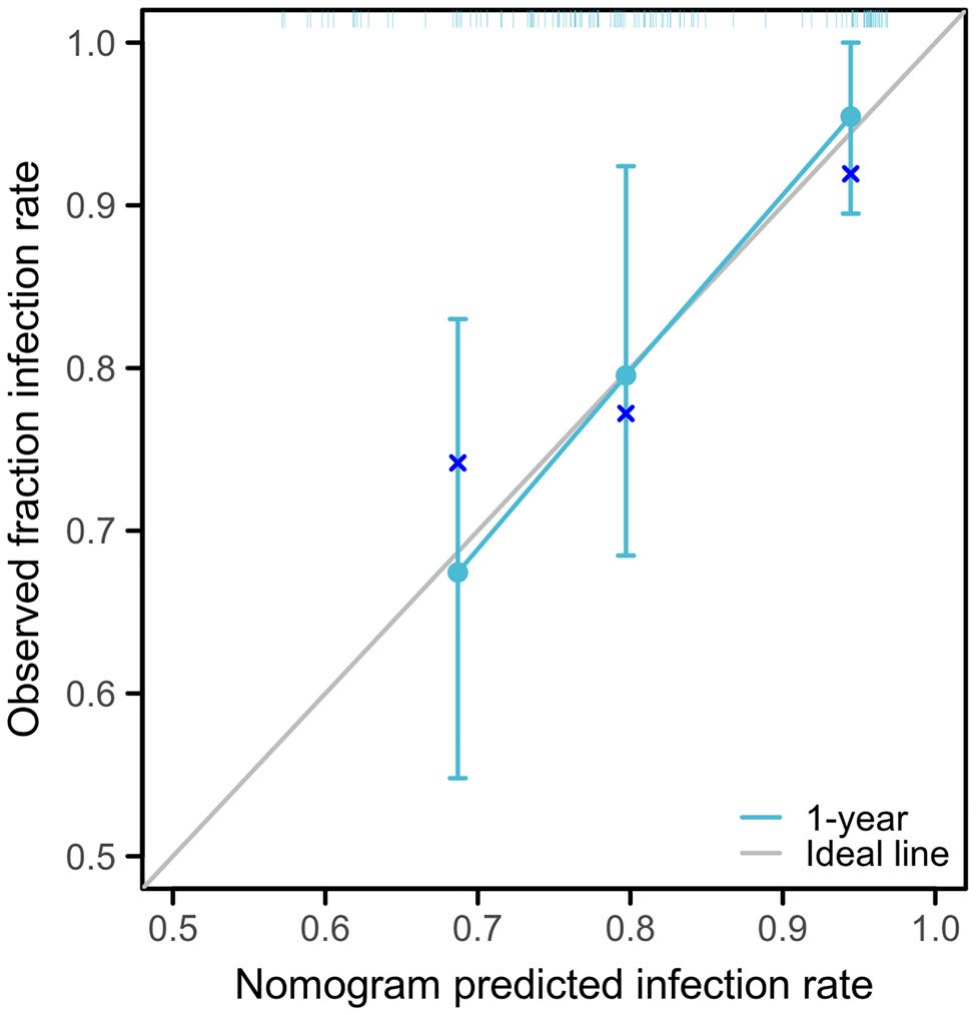
Calibration curves for the prediction of 1-year early urinary tract infection incidence of patients following kidney transplantation.

The risk factors for the early development of UTI following kidney transplantation were evaluated. Variables with *p* < 0.1, including sex and ADR, were involved in the multivariate logistic regression analysis. It was found that ADR was an independent risk factor for the early development of UTI following kidney transplantation (hazard ratio (HR): 5.306, 95% CI: 1.243-22.660, *p* = 0.024) (Table 3), suggesting that a high ADR level increased the risk of UTI by 5.306 times. Using the results of the multivariate logistic regression analysis, a nomogram was drawn to predict the probability of UTI events. The scale score was set and the total score was calculated (Figure 3). To evaluate the fitness of the Cox regression model, another prognostic calibration diagram was drawn (Figure 4). The results indicated that the prediction model exhibited a notable diagonal fit. Acute rejection occurred in 3 and 5 patients in the low ADR and high ADR groups, respectively, without significant differences *(p* > 0.05). None of the other types of rejection occurred in the two groups within one year.

**Table 3.** Cox regression analysis of risk factors for early UTI incidence.

Parameters	Univariate analysis			Multivariate analysis		
	HR	(95%CI)	P	HR	(95%CI)	P
Age	1.006	(0.969–1.045)	0.745			
Gender	0.469	(0.207–1.061)	0.069	0.558	(0.246–1.269)	0.164
Diabetes	1.129	(0.388–3.290)	0.824			
Urinary stent indwelling duration	1.019	(0.532–1.953)	0.954			
ADR	5.774	(1.361–24.495)	0.017	5.306	(1.243–22.660)	0.024
Immunomodulatory regimen	0.996	(0.455–2.184)	0.993			

In addition, CD4+ T cell count was quantified at the time of the UTI episode. A linear relationship was found between CD4+ T cell count and ADR during UTI (*p* = 0.0004, R=-0.668) (Supplementary Fig. S2).

## Discussion

Selecting an optimized dosage of ATG-F for inducing immunologic tolerance of kidney allograft not only is protective against acute rejection, but also confers a lower infection risk. In this study, evidence was provided demonstrating a weight-based ATG-F dosing strategy for reducing the risk of early development of UTIs following kidney transplantation among recipients who were not at high immunologic risk for rejection.

UTI frequently occurs in kidney transplantation recipients. Risk factors for the development of UTI include female gender, advanced age, prior experience of recurrent UTI before receiving transplantation, prolonged urinary catheterization, DD sources of kidney allograft, delayed graft function, and the type of induction and immunomodulatory agents used [2,13–15]. ATG-F is frequently utilized to induce immunologic tolerance during the peri-operative period of kidney transplantation, which could be attributed to its excellent protective effect against rejection and achieving immunologic tolerance [6,16]. However, ATG-F may increase the risk of infection following kidney transplantation [17]. It was previously reported that the total risk of infection and UTI significantly increased among kidney transplant recipients who were given 400-600 mg of ATG-F during the peri-operative period. Postoperative infection has also exhibited to be a potential risk factor for graft loss and recipient mortality [10]. The optimal dose and the frequency of administering ATG-F were further described for induction. Nonetheless, a standardized ATG-F dosing strategy is still unavailable, and there remains a critical need to investigate the appropriate range of ADR in this population [18].

Yilmaz et al. found that when the cumulative ATG-F dose for induction therapy of kidney transplantation was 676 ± 274 mg and the average cumulative dose was 10.6 ± 3.8 mg/kg, the acute rejection incidence was 25.10% (37/147) [19]. However, the present study utilized an average cumulative dose of 458.00 ± 80.98 mg and an average cumulative dose-weight ratio of 7.05 ± 1.53 mg/kg, leading to an acute rejection incidence of only 6.10% (8/131). These findings indicated that there might be flexibility regarding ATG-F dose required to achieve induction and protect against rejection. Du et al. reported in a meta-analysis that when ATG-F was delivered as a single high dose of 9 mg/kg, the incidence of acute rejection could be lowered significantly, while the incidence of UTI could reach 21.67% [20]. Consistently, it was found in the present study that the incidence of UTI in 131 transplant recipients was 19.08%. However, it was noted that when the cumulative ATG-F dose was declined to less than 6.34 mg/kg, the incidence of UTI decreased to as low as 5.00% (2/41). Therefore, lowering the cumulative ATG-F dose with the purpose of reducing infection could be accomplished without increasing the risk of acute rejection among kidney transplant recipients.

Alangaden et al. also revealed that peri-operative ATG-F use for transplantation induction was as an independent risk factor for subsequent infection among kidney transplantation recipients [21]. Pham et al. reached a similar conclusion to that of the present study, in which both studies supported the outcome that perioperative ATG-F could be a significant risk factor for early postoperative UTIs [22]. However, none of the prior studies examined the associations between the risk of early development of UTIs following kidney transplantation and the cumulative ATG-F dose. In the present study, a new method of dosing ATG-F was adopted according to the recipient’s weight. According to the results of the ROC curve analysis, patients were divided into those with low ADR and high ADR, and it was found that the incidence of early postoperative UTI in the low ADR group was significantly lower than that in the high ADR group. Although the reason for this phenomenon remains unknown, an excessive ATG-F dose might reduce absolute T cell count through attenuating nuclear factor-κB (NF-κB) signaling pathway [23]. Prior studies reported that a high ATG-F dose significantly reduced T cell count during early postoperative periods and elevated the incidence of cytomegalovirus infection, partially accounting for their susceptibility to UTIs [24–27]. The future research will concentrate on relevant mechanisms.

The present study had some limitations that should be noted. Firstly, this was a retrospective, single center, and non-randomized study. In addition, social and economic factors might influence the male-to-female ratio of the study participants, leading to an uneven gender ratio. This phenomenon might synergize with other factors and result in selection bias, impacting the study results. Moreover, the anatomic status of the lower urinary tract can also affect the occurrence of UTIs. Most patients with uremia who have been anuric for an extended period may experience varying degrees of bladder contracture. In particular, studies have shown that when the bladder volume is extremely small, the likelihood of infection increases [28]. This study could not completely exclude the influence of anatomical status of the lower urinary tract on the results.

In conclusion, this study revealed that the ratio of cumulative ATG-F dose to kidney transplant recipient weight during the peri-operative period was an independent risk factor for early postoperative UTIs. Optimizing the ADR through dose reduction may benefit kidney transplant recipients by lowering the risk of infection and reducing the likelihood of transplant rejection.

## Supplementary Material

Figure 1.tiff

FigureS1.tif

Figure 4.tiff

Figure 3.tiff

Figure S2.png

Figure 2.tiff

FigureS3.tif

## Data Availability

The datasets used and/or analyzed in the current study are available from the corresponding author upon reasonable request.
