# Supplementary figures and images for: Cumulative rabbit anti-human thymocyte globulin dose to recipient weight during the peri-operative period is an independent risk factor for early postoperative urinary tract infection after kidney transplantation
Source: Ren Fail. 2024 Oct 16;46(2):2414841. doi: 10.1080/0886022X.2024.2414841 (PMC11485816; doi:10.1080/0886022X.2024.2414841)

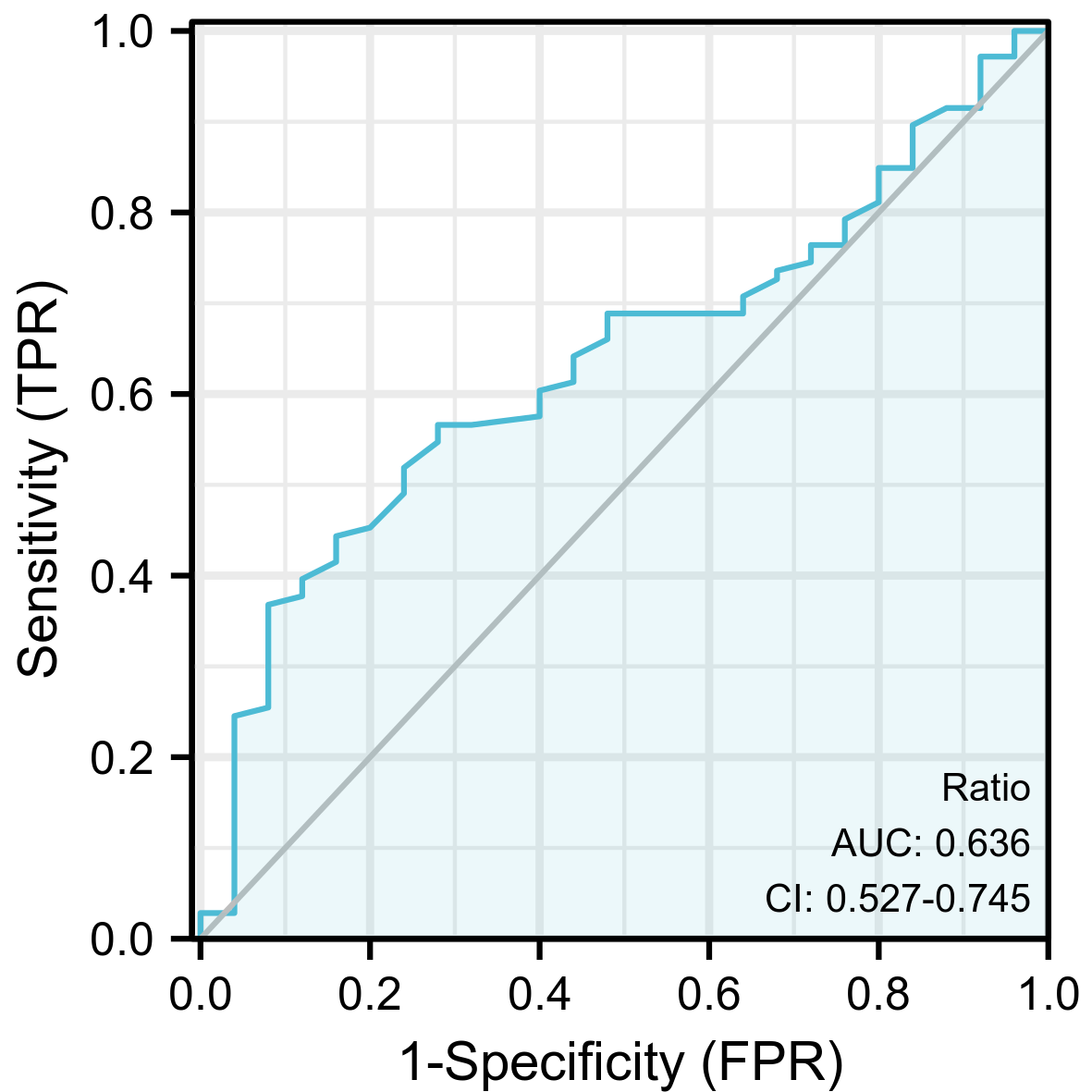

Supplement: Figure 1.tiff [file IRNF_A_2414841_SM7247.tiff]

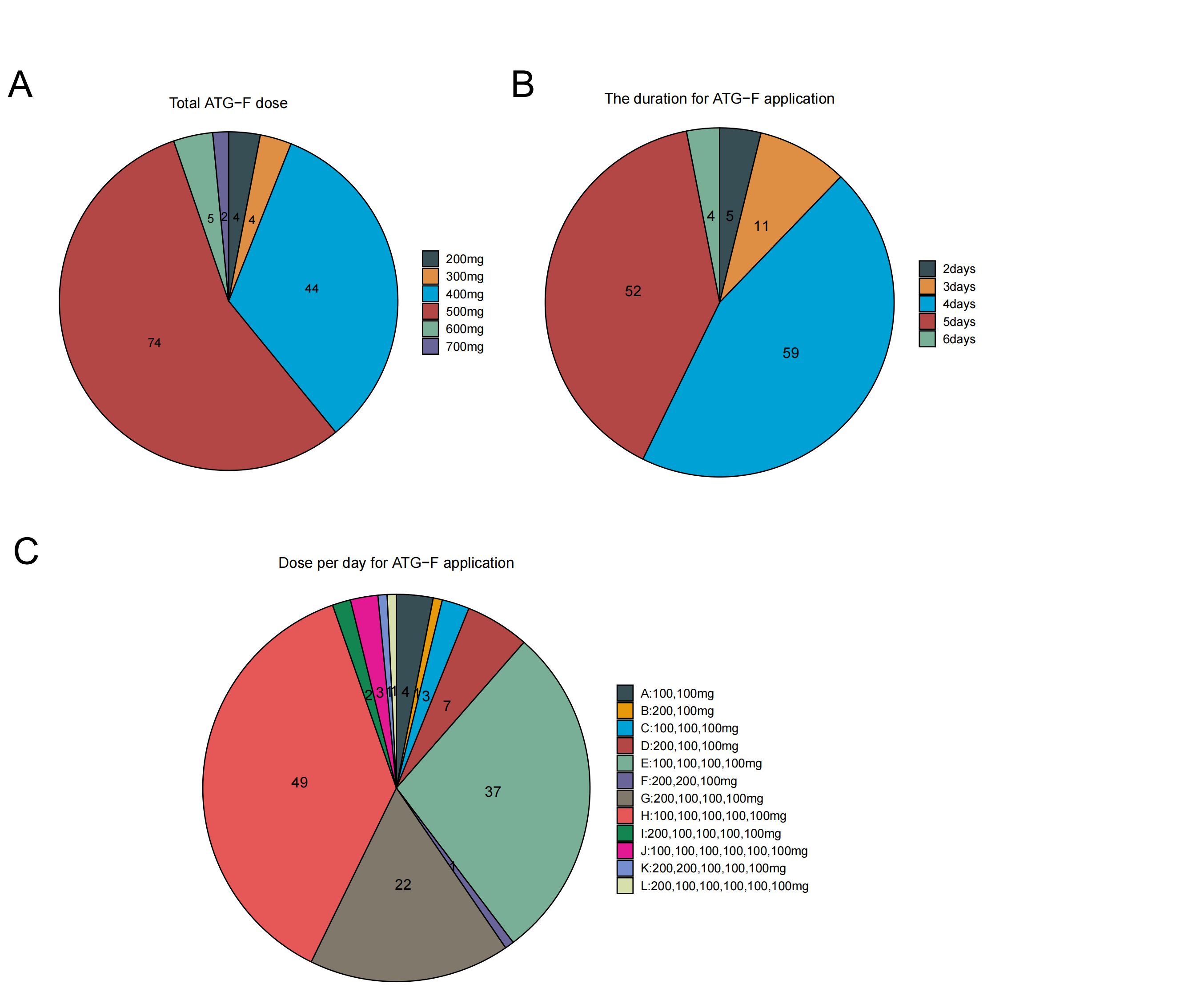

Supplement: FigureS1.tif [file IRNF_A_2414841_SM7246.tif]

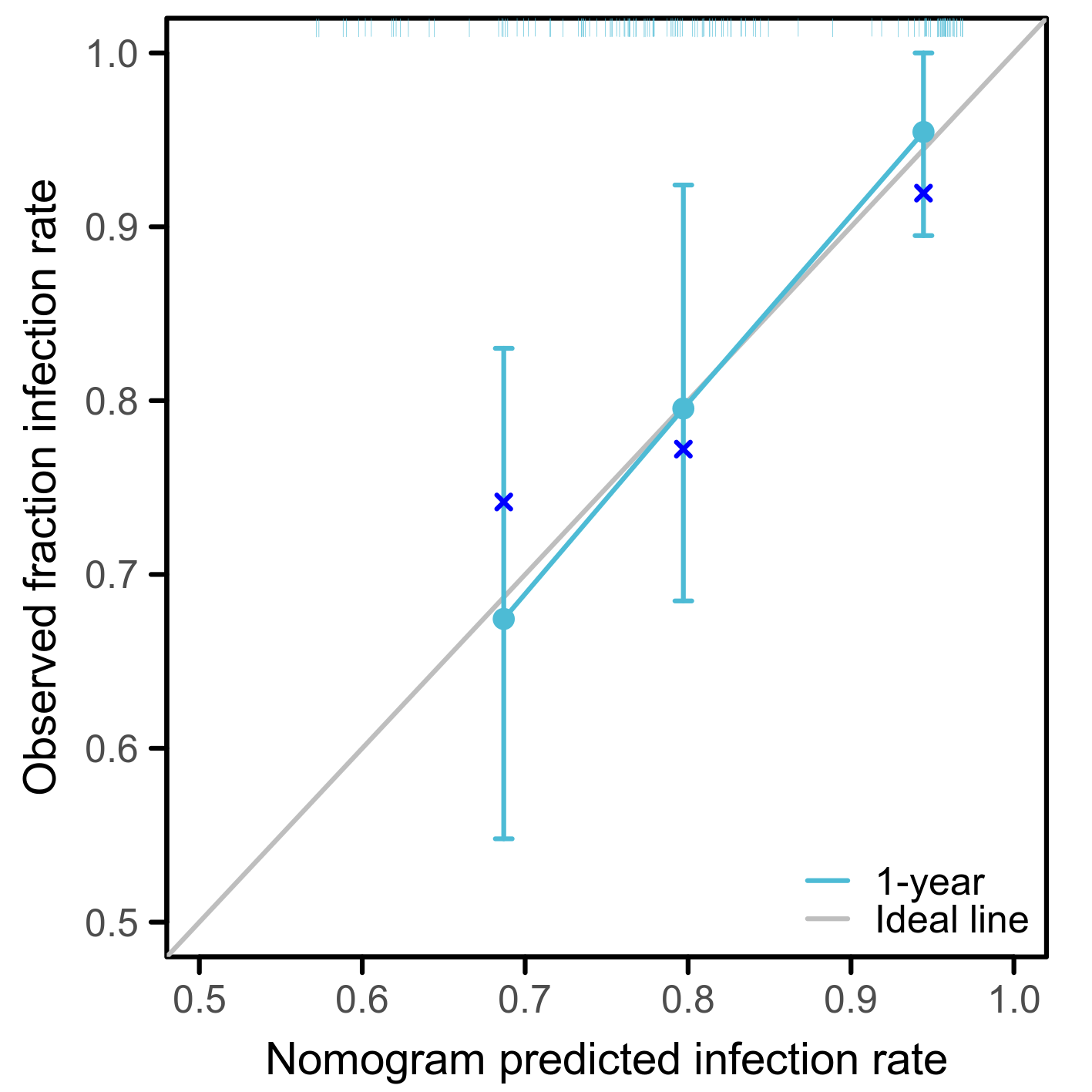

Supplement: Figure 4.tiff [file IRNF_A_2414841_SM7245.tiff]

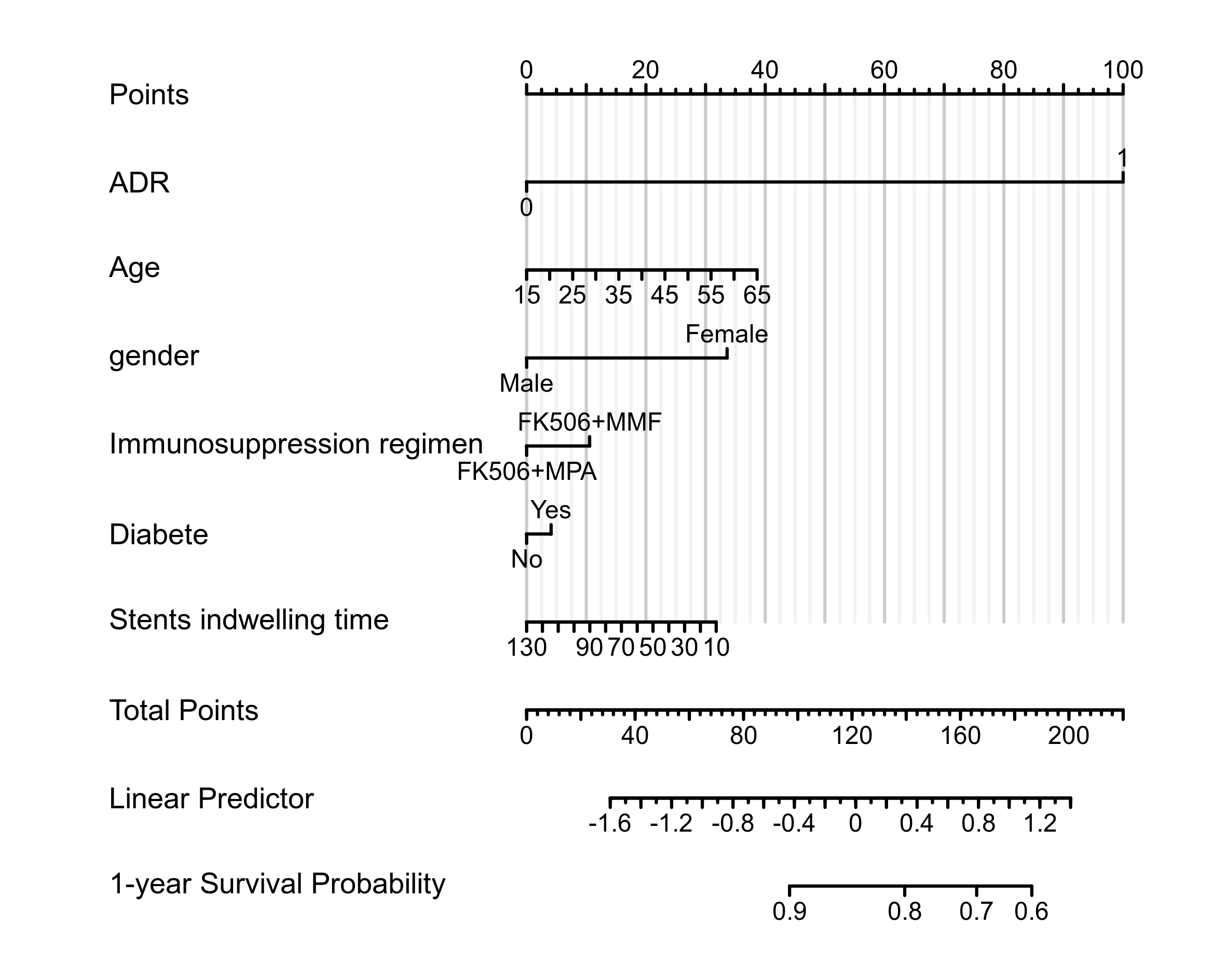

Supplement: Figure 3.tiff [file IRNF_A_2414841_SM7244.tiff]

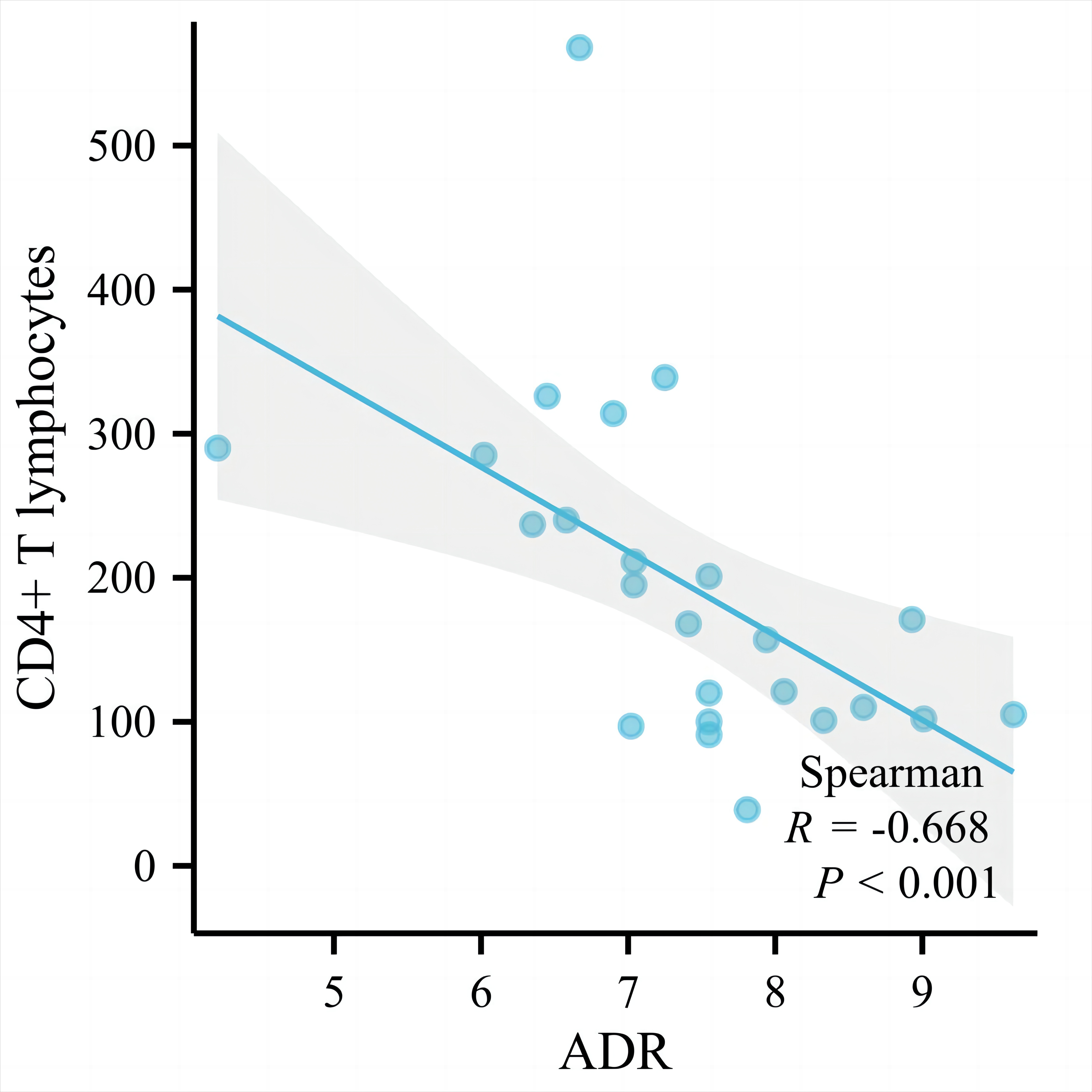

Supplement: Figure S2.png [file IRNF_A_2414841_SM7243.png]

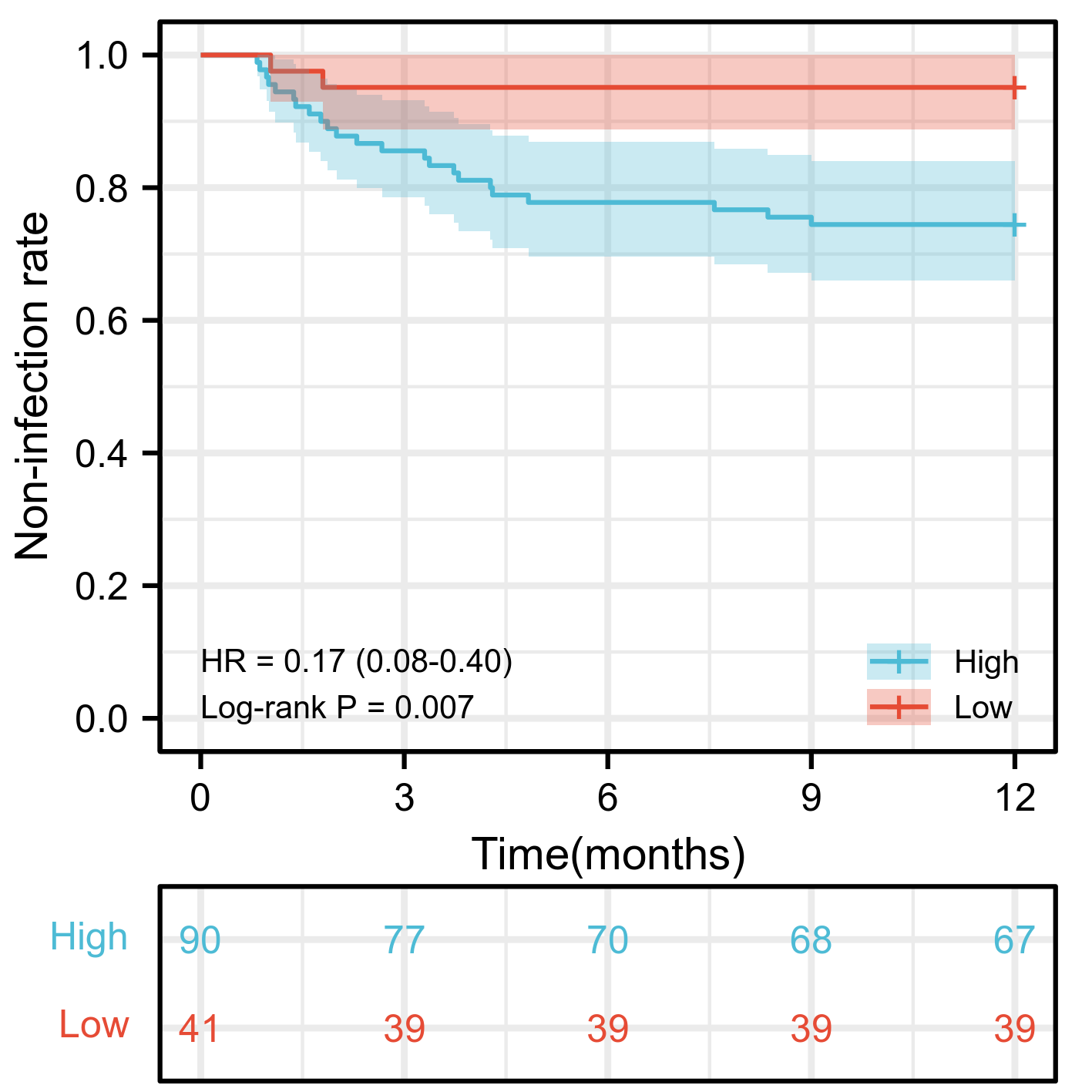

Supplement: Figure 2.tiff [file IRNF_A_2414841_SM7242.tiff]

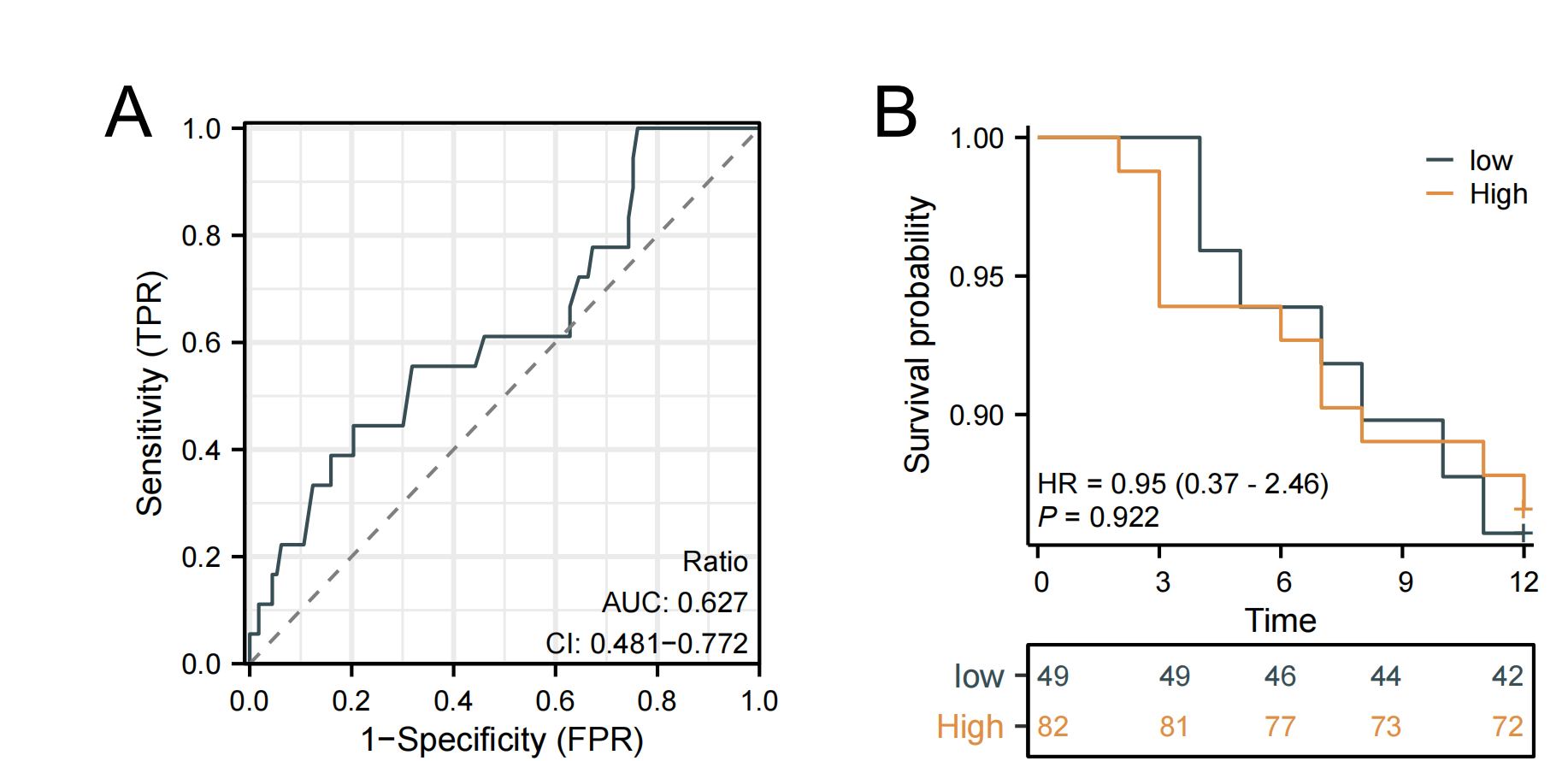

Supplement: FigureS3.tif [file IRNF_A_2414841_SM7241.tif]
